# Variation by lineage in serum antibody responses to influenza B virus infections

**DOI:** 10.1371/journal.pone.0241693

**Published:** 2020-11-09

**Authors:** Yiu Chung Lau, Ranawaka A. P. M. Perera, Vicky J. Fang, Long Hei Luk, Daniel K. W. Chu, Peng Wu, Ian G. Barr, J. S. Malik Peiris, Benjamin J. Cowling

**Affiliations:** 1 World Health Organization Collaborating Centre for Infectious Disease Epidemiology and Control, School of Public Health, The University of Hong Kong, Hong Kong, China; 2 World Health Organization Collaborating Centre for Reference and Research, Melbourne, Victoria, Australia; 3 Department of Microbiology and Immunology, University of Melbourne, Melbourne, Victoria, Australia; 4 HKU-Pasteur Research Pole, School of Public Health, The University of Hong Kong, Hong Kong, China; Public Health England, UNITED KINGDOM

## Abstract

Two lineages of influenza B virus currently co-circulate and have distinct antigenicity, termed Victoria and Yamagata after the B/Victoria/2/87 and B/Yamagata/16/88 strains, respectively. We analyzed antibody titer dynamics following PCR-confirmed influenza B virus infection in a longitudinal community-based cohort study conducted in Hong Kong from 2009–2014 to assess patterns in changes in antibody titers to B/Victoria and B/Yamagata viruses following infections with each lineage. Among 62 PCR-confirmed cases, almost half had undetectable hemagglutination inhibition (HAI) antibody titers to the lineage of infection both pre-infection and post-infection. Among those infected with influenza B/Victoria who showed an HAI titer response after infection, we found strong rises to the lineage of infection, positive but smaller cross-lineage HAI titer boosts, a small dependence of HAI titer boosts on pre-infection titers, and a shorter half-life of HAI titers in adults. Our study is limited by the low HAI sensitivity for non-ether-treated IBV antigen and the incapacity of performing other assays with higher sensitivity, as well as the mismatch between the B/Yamagata lineage circulating strain and the assay strain in one of the study seasons.

## Introduction

Influenza B virus infections are responsible for a considerable fraction of all influenza-associated morbidity and mortality [1]. Two lineages of influenza B virus with distinct antigenicity, termed Victoria and Yamagata after the B/Victoria/2/87 and B/Yamagata/16/88 strains, respectively. They have been co-circulating globally since at least 2001 [1, 2]. A number of studies have noted distinct patterns in age distributions of infections with influenza B/Victoria and B/Yamagata virus strains, with viruses of both lineages being most commonly identified in children, but influenza B/Yamagata virus infections more frequently identified in adults and particularly older adults [3, 4]. One of the possible explanations for this pattern is “imprinting”, where immune responses to recent infections are affected by primary infection in childhood [5]. In this study, we examined changes in serum antibody titers against influenza B virus lineages among PCR-confirmed cases in a community-based cohort study. The objective of our study was to assess patterns in changes in antibody titers to B/Victoria and B/Yamagata viruses following infections with each lineage.

## Materials and methods

### Ethical approval

The study protocol was approved by the Institutional Review Board of the University of Hong Kong. We obtained written consent from participants 18 years of age or older. For participants aged 2 to 7 years, proxy written consent from parents or legal guardians was obtained. For participants aged 8 to 17 years, proxy written consent from parents or legal guardians was obtained in addition to their own written assent.

### Participants

We analyzed data from a community-based cohort study conducted in Hong Kong [6]. The first year of this study was a randomized controlled trial of influenza vaccination, and observational follow-up of participants continued for a further 4 years. Between August 2009 and February 2010, one eligible child 6–17 years of age from each of 796 enrolled households was randomly allocated to receive either a single dose of trivalent inactivated influenza vaccine (TIV) or saline placebo intramuscularly. Serum specimens were collected from every household member at baseline and at 6-month intervals thereafter, for 5 years. We also collected sera 30 days after vaccination from the children who received TIV or placebo in year 1. We contacted families every other week during periods of increased local influenza activity to identify acute respiratory illnesses in household members. If any household member reported 2 or more symptoms or signs of fever ≥37.8°C, chills, headache, sore throat, cough, presence of phlegm, coryza, or myalgia acute, we arranged a home visit to collect a pooled nasal and throat swab for laboratory testing. Since vaccination would induce changes in serum antibody titers, participants who reported receipt of influenza vaccination in a particular period of follow-up would be excluded from analysis for that period.

### Laboratory methods

Serum antibody titers were determined by hemagglutination inhibition (HAI) assays in serial doubling dilutions from an initial dilution of 1:10 using standard methods [7, 8]. In year 1 (2009/10), serum specimens were tested against B/Brisbane/60/2008-like (B/Victoria lineage) and B/Florida/4/2006 (B/Yamagata lineage). In year 2 (2010/11), year 3 (2011/12), year 4 (2012/13) and year 5 (2013/14), serum specimens were tested against the same strains as year 1 for B/Victoria, plus B/Wisconsin/1/2010 (B/Yamagata lineage). HAI titers were the reciprocal of the highest dilution of serum that completely prevented hemagglutination, and titers <10 were imputed as 5. Pooled nose and throat swabs were tested by reverse transcription polymerase chain reaction (PCR) for influenza A and B, and the lineage of influenza B positives was determined with lineage-specific primers [7, 8].

### Statistical analysis

We constructed a mathematical model to describe the short-term increases in HAI titers against B/Victoria and B/Yamagata associated with PCR-confirmed influenza B virus infection (“boosting”) to a peak at 4 weeks after infection, and the longer-term declines in HAI titers (“waning”) beyond 4 weeks [9]. Analyses were stratified by age (≤18 years vs >18 years). All parameters were summarized in Table 1.

**Table 1:** Description of model parameters.

Parameter	Description	Prior
*C*_*j*_	Magnitude of titer boost of lineage *j* in children	Half-Normal(0, 10^2^)
*r*_*j*_	Proportional reduction of magnitude of titer boost of lineage *j* in adults	Uniform(0, 1)
*k*_*j*_	Dependence of magnitude of titer boost of lineage *j* on the pre-infection titer	Gamma(2, 0.1)
*d*_*j*_	Proportional reduction of magnitude of titer boost of lineage *j* during the infection of opposing lineage	Uniform(0, 1)
*q*	Effect of peak titer on age-specific short-term waning rate	Gamma(2, 0.1)
$w_{a_{i}}$	Age-specific rate of titer waning for individual *i*	Gamma(2, 0.1)
*σ*^*obs*^	Standard deviation of measurement error	Half-Normal(0, 10^2^)

Prior to infection, the HAI titer was assumed to wane exponentially along time with an age-specific long-term waning rate as follows:

$$h_{i,j}(t_{i})=h_{i,j}(t_{i}^{pre})\exp\left(-w_{l,a_{i}}∙(t_{i}-t_{i}^{pre})\right),\;t_{i}^{pre}\leq t_{i}\leq t_{i}^{pcr}$$

Herein *t*_*i*_ is time, $t_{i}^{pre}$ is the collection date for the pre-infection serum, and $t_{i}^{pcr}$ is the collection date for the pooled nasal and throat swab for PCR testing. For simplicity, $t_{i}^{pcr}$ is treated as the date of infection. The parameter *h*_*i*,*j*_(*t*) is the titer measure of lineage *j* at time *t*, and $w_{l,a_{i}}$ is the age-specific long-term rate of titer waning where *a*_*i*_ is the age group of individual *i*.

Shortly following PCR-confirmed infection, the HAI titer was assumed to rise, with the magnitude of titer boosting dependent on the age-specific magnitude of titer boost of the corresponding lineage, the pre-infection titer, and the influenza B lineage. Given that the titer values are fold-dilutions {5, 10, 20, 40, …}, we define a log titer (*lh*) for the titer value:

$${lh}_{i,j}(t)=\log_{2}\left(\frac{h_{i,j}(t)}{5}\right)$$

We also specified the age-specific magnitude of titer boost of lineage *j* $\left(C_{a_{i},j}\right)$ as follows:

$$C_{a_{i},j}=\left\{ \begin{array}{rr} C_{j}, & \mathrm{if}\;a_{i}=\mathrm{child}\\ (1-r_{j})C_{j}, & \mathrm{if}\;a_{i}=\mathrm{adult} \end{array}\right.$$

The parameter *C*_*j*_ is the magnitude of titer boost of lineage *j* for children with pre-infection titer <10. It was estimated that the magnitude of titer rises decreased in the age group with the elder individual of influenza A virus infection [10]. Similarly, we propose *r*_*j*_ as the proportional reduction of the magnitude of titer boost of lineage *j* to account for the age effect on the titer rise. Thus, we define the magnitude of titer boost of lineage *j* (*B*_*i*,*j*_) with *I*_*i*_ noting the lineage of infection as follows:

$$B_{i,j}={lh}_{i,j}(t_{i}^{peak})-{lh}_{i,j}(t_{i}^{pcr})=\left\{ \begin{array}{rr} C_{a_{i},j}\exp\left(-k_{j}*{lh}_{i,j}(t_{i}^{pcr})\right), & I_{i}=j\\ (1-d_{j})C_{a_{i},j}\exp\left(-k_{j}*{lh}_{i,j}(t_{i}^{pcr})\right), & I_{i}\neq j \end{array}\right.$$

Herein $t_{i}^{peak}$ is the date for antibody titer reaching the peak, and the magnitude of titer boost is thus equivalent to the difference between peak log titer and the log titer at the date of infection. The constant *k*_*j*_ is to account for the declined magnitude of titer boost of lineage *j* due to high pre-infection titer given the evidence that higher initial titer would lead to smaller titer boost during the infection [11]. The parameter *d*_*j*_ is the proportional reduction of the magnitude of titer boost of lineage *j*, which accounts for the cross-lineage titer boost during the infection of opposing lineage [12], i.e. *I*_*i*_≠*j*.

According to a study of the trajectory of A(H1N1)pdm09 antibody titer [9], the time for antibody titer reaching the peak was 4 weeks after infection assuming that the titer boost followed the gamma density function. We thus fix the time for antibody titer of both lineages reaching the peak to be 4 weeks, and assume that the titers rise following the gamma density function and peak at the mode. For simplicity, we assume the shape parameter (*α*) to be 1.1 and the rate parameter (*β*) to be 1/280 and obtain the mode at 28 days. The gamma density function is then normalized to the range between 0 and 1, representing the proportional titer boost along time as follows:

$$\gamma (t_{i})=\frac{{\left(t_{i}-t_{i}^{pcr}\right)}^{\alpha -1}\exp\left(-\beta (t_{i}-t_{i}^{pcr})\right)}{{\left(t_{i}^{peak}-t_{i}^{pcr}\right)}^{\alpha -1}\exp\left(-\beta (t_{i}^{peak}-t_{i}^{pcr})\right)},\;t_{i}^{pcr}<t_{i}\leq t_{i}^{peak}$$

Given the magnitude of titer boost and the function of titer boost over time, the titer would rise until the time of peak titer $\left(t_{i}^{peak}\right)$ following the function below:

$$h_{i,j}(t_{i})=h_{i,j}(t_{i}^{pcr})∙2^{\left(B_{i,j}∙\gamma (t_{i})\right)},\;t_{i}^{pcr}<t_{i}\leq t_{i}^{peak}$$

After reaching the peak titer, we allowed for the possibility that the HAI titer would wane exponentially at a faster rate within the first 6 months [9]. This age-specific short-term waning rate was assumed to be a function of the peak titer, the age-specific long-term waning rate, and time. After 6 months, the HAI titer would then decline over time with the same long-term waning rate as prior to infection. The age-specific short-term waning rate is thus assumed to be a function of the peak titer and age-specific long-term waning rate as follows:

$$w_{s,a_{i}}=w_{l,a_{i}}+q∙\max\left(0,{lh}_{i,j}(t_{i}^{peak})\right)$$

The parameter *q* is the effect of peak titer on the age-specific short-term waning rate. It is also assumed that the waning rate would decline linearly from $w_{s,a_{i}}$ to $w_{l,a_{i}}$ within 6 months, and afterwards the titer would wane over time with the age-specific long-term waning rate $w_{l,a_{i}}$ as prior to infection at time $t_{i}^{long}$. The function of the waning rate at time *t*_*i*_ is shown below:

$$w_{i}(t_{i})=\left\{ \begin{array}{c} w_{l,a_{i}}+(w_{s,a_{i}}-w_{l,a_{i}})∙\frac{t_{i}^{long}-t_{i}}{t_{i}^{long}-t_{i}^{peak}}\;,\;t_{i}^{peak}\leq t_{i}\leq t_{i}^{long}\\ w_{l,a_{i}}\;,\;t_{i}>t_{i}^{long} \end{array}\right.$$

The trajectory of titer waning after reaching the peak titer before the collection date of post-infection serum, $t_{i}^{post}$, is described as follows:

$$h_{i,j}(t_{i})=h_{i,j}(t_{i}^{peak})\exp\left(-\int_{t_{i}^{peak}}^{t_{i}}t∙w_{i}(t)dt\right),\;t_{i}^{peak}<t_{i}\leq t_{i}^{post}$$

For the individual who did not have any PCR-confirmed infection, they were assumed to have no infection in the past 6 months and the HAI titer would only wane over time with the age-specific long-term waning rate:

$$h_{i,j}(t_{i})=h_{i,j}(t_{i}^{pre})\exp\left(-w_{a_{i}}∙(t_{i}-t_{i}^{pre})\right),\;t_{i}^{pre}\leq t_{i}\leq t_{i}^{post}$$

We assume the observed log titer of lineage *j* of individual *i* at time $t_{i}^{post},\;{lh}_{i,j}^{obs}(t_{i}^{post})$, is normally distributed with the latent log titer ${lh}_{i,j}(t_{i}^{post})=\mu_{i,j}$ as mean and *σ*^*obs*^ as the standard deviation of the measurement error. The likelihood of observing ${lh}_{i,j}^{obs}(t_{i}^{post})=\lambda_{i,j}$ would be as follows:

$$L(\lambda_{i,j}|\mu_{i,j},\sigma^{obs})=\left\{ \begin{array}{cc} \Phi (1|\mu_{i,j},\sigma^{obs}) & 1>\lambda_{i,j}\\ \Phi (\lambda +1|\mu_{i,j},\sigma^{obs})-\Phi (\lambda |\mu_{i,j},\sigma^{obs}) & 1\leq \lambda_{i,j} \end{array}\right.$$

where Φ(*x*|*μ*,*σ*) is the cumulative normal distribution with percentile *x*, mean *μ* and standard deviation *σ*.

The posterior distribution of the parameters was obtained by using the Hamiltonian Monte Carlo algorithm in RStan package version 2.19.2 [13]. This was implemented with 4 chains, each with 7000 iterations including 2000 iterations in the warm-up period. The convergence of posterior distribution would be visually assessed and diagnosed by the built-in HMC Diagnostics in RStan package. All statistical analyses were conducted in R version 3.5.1 (R Foundation for Statistical Computing, Vienna, Austria) [14].

### Inclusion criteria

We set the following inclusion criteria for analyses. Participants with PCR-confirmed influenza B virus infection would be included in analyses, and we examined patterns in B/Victoria and B/Yamagata titers for infections with both lineages, i.e. same strain titer responses and also cross-reactions. In a secondary analysis, we excluded individuals who had pre-infection and post-infection titers <10 for the infecting lineage. Participants who did not have any PCR-confirmed infection were included in the analysis to inform long-term waning rates if their pre-season HAI titers of both lineages were ≥10 and greater than or equal to the corresponding post-season titers.

### Sensitivity analysis

The time for antibody titer reaching the peak (*t*^*peak*^) and the period for short-term waning rate (*t*^*long*^) were fixed to be 4 weeks and 6 months after infection in the model. We thus conducted a sensitivity analysis on different *t*^*peak*^ and *t*^*long*^ based on the data for sub-analysis as the PCR-confirmed cases showing antibody response would be more sensitive to changes in the model. We would compare the estimates when *t*^*peak*^ was 2 weeks and 8 weeks, and when *t*^*long*^ was 3 months and 9 months. We also assessed the model adequacy by comparing the observed and the posterior post-infection HAI titer.

## Results

During the study period between August 2009 and December 2014, we identified 35 PCR-confirmed influenza B/Victoria lineage infections and 27 infections of B/Yamagata lineage (Table 2 and S1 Table). Post-infection sera were collected a mean of 6 months after the PCR confirmation (range 26–321 days). Most of the cases were in children, including 30/35 (86%) of the B/Victoria lineage infections and 17/27 (63%) of the B/Yamagata infections, and the median age was 10 for both lineages.

**Table 2:** Characteristics of the PCR-confirmed cases.

	B/Victoria lineage		B/Yamagata lineage	
	Overall	Sub-analysis	Overall	Sub-analysis
PCR-confirmed cases	35	16	27	15
Age				
Children	30 (86)	16 (100)	17 (63)	10 (67)
Adult	5 (14)	0 (0)	10 (37)	5 (33)
Time from PCR sampling to post-season sampling (days)				
≤6 months	15 (43)	9 (56)	11 (41)	6 (40)
>6 months	20 (57)	7 (44)	16 (59)	9 (60)
Pre-B/Victoria HAI^a^ titer				
<10	34 (97)	15 (94)	23 (85)	11 (73)
10–20	1 (3)	1 (6)	2 (7)	2 (13)
≥40	0 (0)	0 (0)	2 (7)	2 (13)
Post-B/Victoria HAI^a^ titer				
<10	19 (54)	0 (0)	19 (70)	10 (67)
10–20	5 (14)	5 (31)	2 (7)	0 (0)
≥40	11 (31)	11 (69)	6 (22)	5 (33)
Pre-B/Yamagata HAI^a^ titer				
<10	25 (71)	11 (69)	27 (100)	15 (100)
10–20	1 (3)	0 (0)	0 (0)	0 (0)
≥40	9 (26)	5 (31)	0 (0)	0 (0)
Post-B/Yamagata HAI^a^ titer				
<10	20 (57)	7 (44)	12 (44)	0 (0)
10–20	2 (6)	1 (6)	6 (22)	6 (40)
≥40	13 (37)	8 (50)	9 (33)	9 (60)

Characteristics of the PCR-confirmed cases in the overall analysis and that of the PCR-confirmed cases showing titer rise against the lineage of infection in the sub-analysis.

^a^ Abbreviation: HAI = hemagglutination inhibition assay.

**Fig 1:**
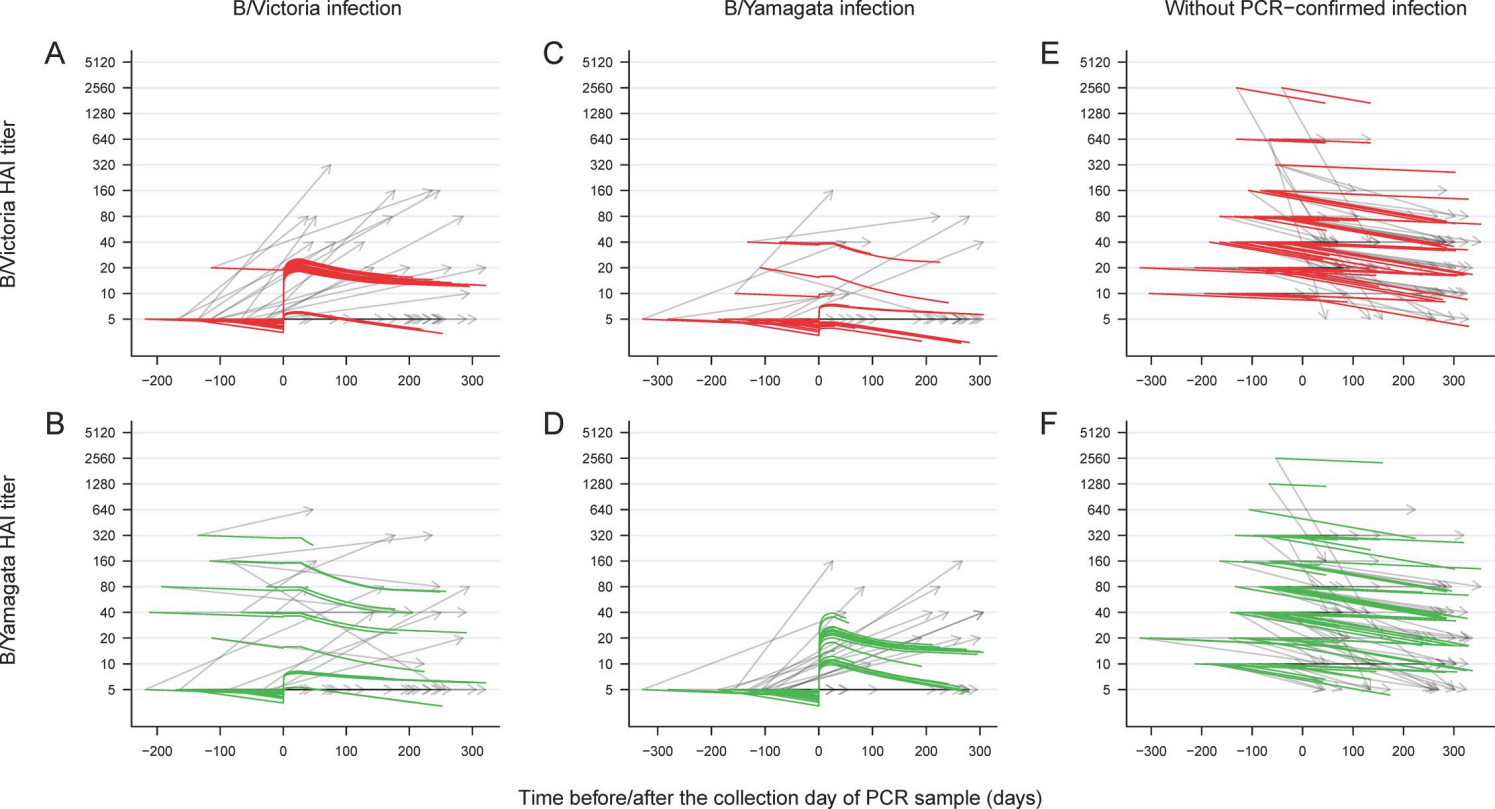
Modeled HAI antibody titer dynamics for the participants with and without PCR-confirmed infection in the overall analysis. Antibody titers measured by the hemagglutination inhibition (HAI) assay against B/Victoria and B/Yamagata lineage viruses (black lines) versus modeled HAI antibody titer dynamics (colored lines) for the PCR-confirmed cases and participants without PCR-confirmed infection from August 2009 to December 2014 included in the overall analysis.

We first conducted an overall analysis with all PCR-confirmed cases, and the HAI titer trajectories along time of whom were illustrated in Fig 1. We estimated that infections with B/Victoria and B/Yamagata led to mean fold rises of 4.4 (95% credibility interval, CrI: 2.0–9.3) and 5.7 (95% CrI: 2.4–12.4) in titers against the corresponding lineage, respectively. Children were estimated to have a slower rate of long-term waning in antibody titer than adults, where the median times for antibody titer to drop by half were 3.4 years and 0.8 years on average, for children and adults, respectively. We also examined cross-reactive responses, finding that infections with B/Victoria led to mean fold rises of B/Yamagata titers of 1.7 (95% CrI: 1.0–3.3), while infections with B/Yamagata led to mean fold rises of B/Victoria titers of 1.6 (95% CrI: 1.0–3.2). Adults had lower mean fold rises than children, for both B/Victoria (60% lower; 95% CrI: 18%-85%) and B/Yamagata (51% lower; 95% CrI: 8%-83%). Moreover, the mean fold rises in titer following B/Victoria and B/Yamagata infections both depended on the pre-infection titer. For B/Victoria infections, the modelled mean fold rise from a pre-infection titer of <10 was 4.0 (95% CrI: 1.7–8.8) for children and 1.4 (95% CrI: 1.0–3.0) for adults, compared to a mean fold rise of 1.3 (95% CrI: 1.0–3.4) and 1.1 (95% CrI: 1.0–1.5) for a pre-infection titer of 40 in children and adults, respectively. For B/Yamagata infections, the modelled mean fold rise from a pre-infection titer of <10 was 4.6 (95% CrI: 1.7–10.9) and 2.0 (95% CrI: 1.0–4.5) in children and adults, compared to a mean fold rise of 1.2 (95% CrI: 1.0–3.2) and 1.1 (95% CrI: 1.0–2.0) for a pre-infection titer of 40 in children and children, respectively.

**Fig 2:**
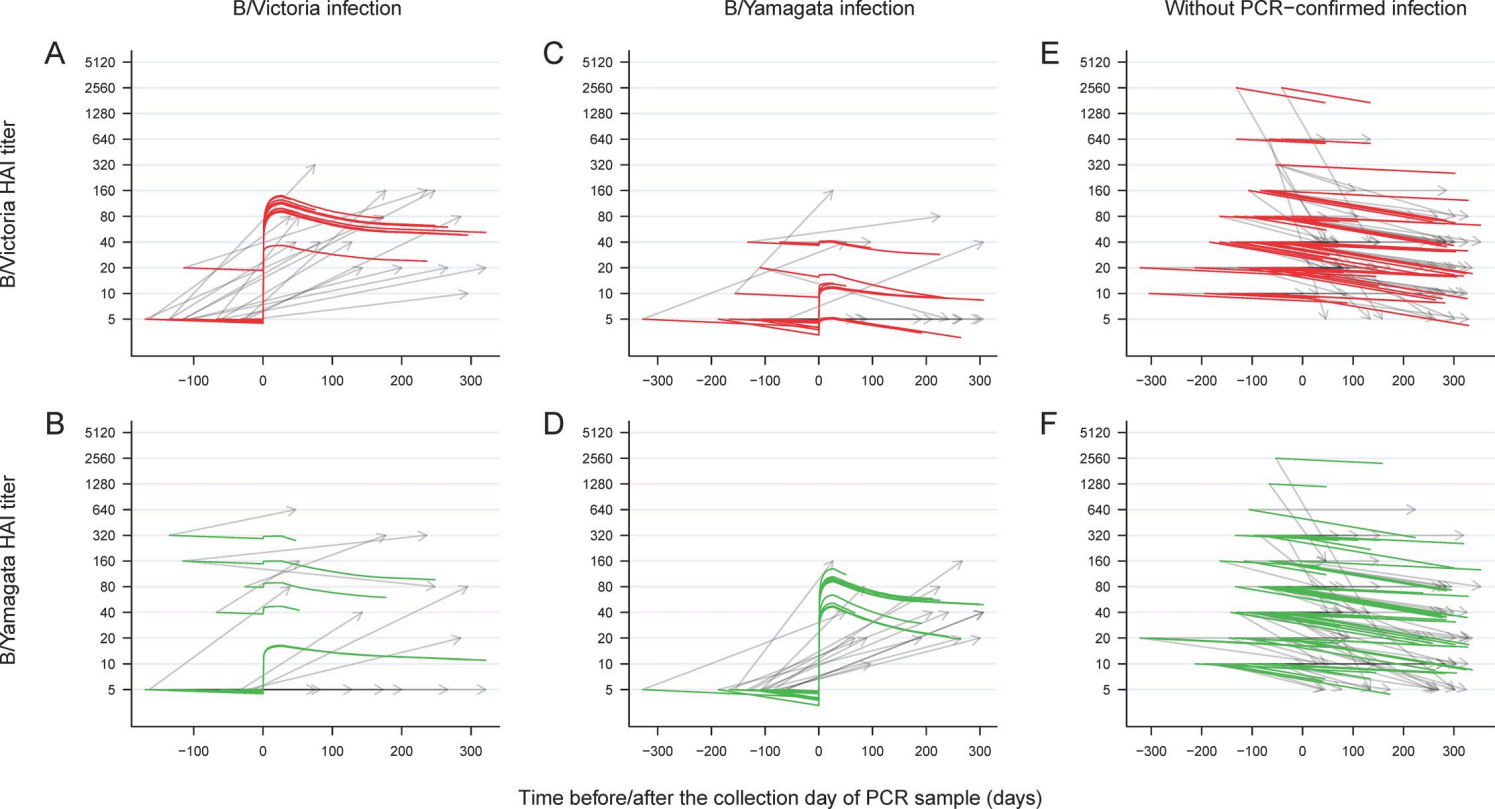
Modeled HAI antibody titer dynamics for the participants with and without PCR-confirmed infection in the sub-analysis. Antibody titers measured by the hemagglutination inhibition (HAI) assay against B/Victoria and B/Yamagata lineage viruses (black lines) versus modeled HAI antibody titer dynamics (coloured lines) for PCR-confirmed cases and participants without PCR-confirmed infection from August 2009 to December 2014 included in the sub-analysis.

However, approximately half of the cases had both pre-infection and post-infection HAI titers <10 against the lineage of infection (B/Victoria: 19/35, 54%; B/Yamagata: 12/27, 44%), from whom we did not see a systematic deviation from the overall characteristic of cases with infection of the corresponding lineage (Table 2). This suggested that the distribution of titer rise was bimodal, leading to the strength of titer rises being underestimated among those who showed a titer rise against the lineage of infection. We thus conducted a sub-analysis excluding the 19 B/Victoria and 12 B/Yamagata cases with pre-infection and post-infection titers <10 (Fig 2), and found that infections with B/Victoria led to mean fold rises in B/Victoria titers of 19.5 (95% CrI: 5.6–48.0) in children, and infections with B/Yamagata led to mean fold rises in B/Yamagata titers of 20.0 (95% CrI: 6.4–50.4) in children. In terms of cross-reactions, infections with B/Victoria led to mean fold rises of B/Yamagata titers of 3.7 (95% CrI: 1.3–9.0), while infections with B/Yamagata led to mean fold rises of B/Victoria titers of 2.8 (95% CrI: 1.0–7.7). Additionally, adults had a 79% (95% CrI: 19%-97%) lower mean fold rise in B/Victoria titers in infection with B/Victoria lineage, and a 54% (95% CrI: 6%-88%) lower mean fold rise in B/Yamagata titers in infection with B/Yamagata lineage. As in the main analysis reported above, geometric mean titer rises were reduced in individuals with higher pre-infection titers, and there were similar estimates of long-term waning rates in antibody in the sub-analysis. The median times for antibody titer to drop by half were 2.9 years in children and 0.8 years in adult on average, respectively.

In the sensitivity analysis, we obtained similar estimates of mean fold rise in titer under different *t*^*peak*^ and *t*^*long*^, suggesting the estimates were not very sensitive to the assumption on *t*^*peak*^ and *t*^*long*^ (S2 Table). Besides, the credible intervals of post-infection titer could cover ≥95% of the observed one by lineage in both main analysis and sub-analysis (S3 Table), suggesting a reasonable model fit for the data.

## Discussion

We integrated longitudinal serological data on PCR-confirmed cases with a statistical model to estimate the antibody titer dynamics following influenza B virus infection. We found that half of PCR-confirmed cases had no detectable rise in HAI titers against the infecting strain. Among those cases where there was a detectable rise in HAI titer, boosts were on average substantial, and higher on average in children than adults. We also identified rises in cross-lineage HAI titers. A systematic review found that trivalent inactivated vaccine could confer partial cross-lineage protection of 42–52%, compared with 65–83% protection for the same lineage included in the vaccine [15]. Our findings on increases in HAI titers of the opposing lineage would also be consistent with some degree of cross-lineage protection.

Age plays an important role in the antibody response against influenza B virus infection. We observed reduced titer boosts in adults and found that the mean fold rise in titer against B/Yamagata lineage was higher than that against B/Victoria lineage in adult in the sub-analysis (Figs 1 and 2). The reduced HAI titer boost might be associated with pre-existing immunity in adults with previous exposure, and the contrast in mean fold rise between lineages was consistent with a previous study that reported boosted antibody responses against B/Yamagata lineage but little against B/Victoria lineage in primed children [12]. We did not examine responses to antibody titers against historical influenza B viruses, but further work could examine whether this phenomenon of differential responses to B/Victoria and B/Yamagata was associated with antigenic imprinting where the imprint established by historical infecting strains would govern the antibody responses to subsequent infections [5]. The imprinting effect might lead to the recall of immunity against B/Yamagata lineage virus in children primed with B/Yamagata lineage even when challenged by B/Victoria lineage virus, hence the boosted response in B/Yamagata HAI titer observed in the previous study. This could confer protection against infection with the influenza strain under the lineage responsible for the imprinting [16]. In addition we estimated more rapid declines in HAI titers in adults than children, consistent with our previous study [9].

Serological response with ≥4-fold rise in HAI titers is used to identify recent infections. However, this criteria may overlook infected cases with high pre-infection titer given limited fold rise in titer during infection due to the ceiling effect [11]. Besides, as suggested in the sub-analysis, the 95% CrI for the cross-lineage response covered values above 4, which implied that the infections of a certain influenza B lineage might lead to a cross-lineage reaction with >4-fold rise in titer of the opposing lineage and hence caused a misclassification of infection with the opposing lineage. The sensitivity and specificity of 4-fold rises in HAI titer for identifying potential influenza B virus infection worthy of further research, while PCR remains the golden standard to determine the infection status and the lineage of infection.

This study strengthened the evidence of cross-lineage immune response against IBV, which might explain the cross-lineage protection against IBV infection. The mismatch between circulating lineage and vaccine lineage in TIV is common [17]. Though the mechanism of the cross-lineage response in HAI titer is yet confirmed, further research might be beneficial to optimize the vaccine component in TIV which contribute a higher degree of cross-lineage protection and thus help to alleviate the disease burden brought by lineage mismatch.

There were some limitations in this study. First, we did not observe HAI titer rises in some infected individuals, suggesting the possibility that our HAI assay was not fully capturing the antibody response to a laboratory-confirmed infection, which could be mediated through other pathways like neuraminidase. Even the antibodies which target HA stalk showed cross-lineage reactivity against both linage viruses given the highly conserved HA stalks [18], such non-HAI-active response cannot be captured by the HAI assay as well. We also found a slight mismatch in B/Yamagata lineage between the predominant strain (B/Massachusetts/2/2012) and the tested strain (B/Wisconsin/1/2010) in year 5, which might contribute to the no-response in HAI titer. Additional serologic analysis with a panel of influenza B viruses could allow a landscape analysis of immune responses to historical as well as contemporary strains. Second, our antigens were not ether split, while using ether-treated antigen might enhance the sensitivity of the HAI assay [19]. A study also found low sensitivity of the HAI assay to influenza B strains, while single radial hemolysis (SRH) or Enzyme-linked immunosorbent assays (ELISAs) could be an alternative for measuring antibody response with a higher sensitivity and specificity [20]. Finally, our study was limited by the small sample size of confirmed cases, which is a challenge for many community-based studies.

In conclusion, we estimated the strength of changes in HAI titers to the homologous and the heterologous influenza B virus lineage for laboratory-confirmed influenza B virus infections, and showed that the changes in HAI titer were associated with several factors like age and pre-infection titer. Further work could examine the translation of cross-lineage HAI titer response to the degree of cross-immunity between influenza B lineages, and how this contributes to epidemiological interactions between the two lineages and their transmission dynamics.

## Supporting information

S1 Table HAI titers in patients with all PCR-confirmed influenza B virus infection. The fold change for the patient with both pre-infection and post-infection sera titers <10 would be denoted as “NA”.
(DOCX)

S2 Table Sensitivity analysis on different time for antibody titer reaching the peak and different period for short-term waning rate. The time for antibody titer reaching the peak and the period for short-term waning rate were fixed to be 4 weeks and 6 months after infection in the model. This table compared the estimates when the time for antibody titer reaching the peak was 2 weeks and 8 weeks, and when the period for short-term waning rate was 3 months and 9 months.
(DOCX)

S3 Table Model adequacy for fitting the data. This table showed the proportion of observation which lied inside the 95% credible intervals of the post-infection titer by lineage of PCR-confirmed infection and lineage titer.
(DOCX)
