# Supplementary material for: Variation by lineage in serum antibody responses to influenza B virus infections
Source: PLoS One. 2020 Nov 9;15(11):e0241693. doi: 10.1371/journal.pone.0241693 (PMC7652285; doi:10.1371/journal.pone.0241693)
Supplement: S1 Table — The fold change for the patient with both pre-infection and post-infection sera titers <10 would be denoted as “NA”. (DOCX) [file pone.0241693.s001.docx]

**Table S1.** **HAI titers in patients with all PCR-confirmed influenza B virus infection.** Children and adult corresponded to the age group ≤18 and >18, respectively. The fold change for the patient with both pre-infection and post-infection sera titers <10 would be denoted as “NA”.

|  |  |  | Days relative to time of infection | | HAI titers against B/Victoria lineage | | | HAI titers against B/Yamagata lineage | | |
| --- | --- | --- | --- | --- | --- | --- | --- | --- | --- | --- |
| Patient (age group/sex) | Date (mm-yyyy) of infection | Lineage of infection | Pre-infection serum | Post-infection serum | Pre-infection serum | Post-infection serum | Fold change | Pre-infection serum | Post-infection serum | Fold change |
| AA (children/ F) | 03-2010 | Victoria | -135 | 47 | <10 | 40 | >4 | 320 | 640 | 2 |
| AB (adult/ F) | 03-2010 | Victoria | -153 | 218 | <10 | <10 | NA | <10 | <10 | NA |
| AC (children/ F) | 05-2010 | Victoria | -26 | 177 | <10 | 160 | >16 | 80 | 320 | 4 |
| AD (children/ M) | 03-2010 | Victoria | -116 | 234 | <10 | <10 | NA | <10 | <10 | NA |
| AE (children/ F) | 03-2010 | Victoria | -114 | 236 | 20 | 160 | 8 | 160 | 320 | 2 |
| AF (children/ F) | 05-2010 | Victoria | -193 | 205 | <10 | <10 | NA | 80 | 40 | -2 |
| AG (children/ F) | 03-2010 | Victoria | -116 | 248 | <10 | 160 | >16 | 160 | 80 | -2 |
| AH (children/ M) | 05-2010 | Victoria | -170 | 173 | <10 | 80 | >8 | <10 | <10 | NA |
| AI (children/ F) | 03-2010 | Victoria | -84 | 257 | <10 | <10 | NA | 160 | <10 | < -16 |
| AJ (children/ F) | 02-2010 | Victoria | -46 | 290 | <10 | <10 | NA | 40 | 40 | 1 |
| AK (adult/ F) | 03-2010 | Victoria | -113 | 223 | <10 | <10 | NA | 20 | 10 | -2 |
| AL (children/ M) | 01-2010 | Victoria | -13 | 295 | <10 | 10 | >1 | <10 | 80 | >8 |
| AM (children/ F) | 04-2010 | Victoria | -132 | 211 | <10 | <10 | NA | <10 | <10 | NA |
| AN (children/ F) | 03-2010 | Victoria | -67 | 52 | <10 | 80 | >8 | 40 | 160 | 4 |
| AO (adult/ F) | 03-2010 | Victoria | -101 | 207 | <10 | <10 | NA | <10 | <10 | NA |
| AP (children/ F) | 03-2010 | Victoria | -23 | 285 | <10 | 80 | >8 | <10 | 20 | >2 |
| AQ (children/ F) | 03-2010 | Victoria | -52 | 235 | <10 | <10 | NA | <10 | <10 | NA |
| AR (children/ M) | 05-2010 | Victoria | -219 | 159 | <10 | <10 | NA | <10 | 80 | >8 |
| AS (children/ M) | 05-2010 | Victoria | -212 | 180 | <10 | <10 | NA | 40 | 40 | 1 |
| AT (children/ F) | 03-2010 | Victoria | -129 | 46 | <10 | <10 | NA | <10 | <10 | NA |
| AU (children/ M) | 04-2010 | Victoria | -106 | 200 | <10 | 20 | >2 | <10 | <10 | NA |
| AV (adult/ F) | 01-2012 | Victoria | -95 | 87 | <10 | <10 | NA | <10 | <10 | NA |
| AW (children/ F) | 01-2012 | Victoria | -66 | 109 | <10 | <10 | NA | <10 | <10 | NA |
| AX (children/ M) | 03-2012 | Victoria | -164 | 39 | <10 | <10 | NA | <10 | <10 | NA |
| AY (children/ F) | 03-2012 | Victoria | -164 | 39 | <10 | 80 | >8 | <10 | 80 | >8 |
| AZ (children/ M) | 03-2012 | Victoria | -135 | 75 | <10 | 320 | >32 | <10 | <10 | NA |
| BA (children/ M) | 03-2012 | Victoria | -105 | 266 | <10 | 20 | >2 | <10 | <10 | NA |
| BB (children/ F) | 01-2012 | Victoria | -72 | 306 | <10 | <10 | NA | <10 | <10 | NA |
| BC (children/ F) | 01-2012 | Victoria | -61 | 128 | <10 | 40 | >4 | <10 | <10 | NA |
| BD (adult/ M) | 02-2012 | Victoria | -80 | 252 | <10 | <10 | NA | <10 | <10 | NA |
| BE (children/ M) | 02-2012 | Victoria | -116 | 248 | <10 | <10 | NA | <10 | <10 | NA |
| BF (children/ F) | 02-2012 | Victoria | -114 | 82 | <10 | 40 | >4 | <10 | <10 | NA |
| BG (children/ M) | 12-2011 | Victoria | -60 | 321 | <10 | 20 | >2 | <10 | <10 | NA |
| BH (children/ M) | 02-2012 | Victoria | -87 | 250 | <10 | <10 | NA | <10 | 40 | >4 |
| BI (children/ F) | 12-2013 | Victoria | -32 | 143 | <10 | 20 | >2 | <10 | 40 | >4 |
|  |  |  | Days relative to time of infection | | HAI titers against B/Victoria lineage | | | HAI titers against B/Yamagata lineage | | |
| Patient (age/sex) | Date (dd-mm-yyyy) of infection | Lineage of infection | Pre-infection serum | Post-infection serum | Pre-infection serum | Post-infection serum | Fold change | Pre-infection serum | Post-infection serum | Fold change |
| BJ (adult/ M) | 07-2010 | Yamagata | -87 | 109 | <10 | <10 | NA | <10 | <10 | NA |
| BK (children/ F) | 01-2010 | Yamagata | -13 | 295 | <10 | <10 | NA | <10 | <10 | NA |
| BL (adult/ NA) | 02-2012 | Yamagata | -126 | 67 | <10 | <10 | NA | <10 | 20 | >2 |
| BM (children/ F) | 02-2012 | Yamagata | -106 | 84 | <10 | <10 | NA | <10 | 80 | >8 |
| BN (adult/ F) | 01-2012 | Yamagata | -101 | 89 | <10 | <10 | NA | <10 | 20 | >2 |
| BO (children/ F) | 03-2012 | Yamagata | -175 | 52 | <10 | <10 | NA | <10 | <10 | NA |
| BP (children/ F) | 01-2012 | Yamagata | -83 | 267 | <10 | <10 | NA | <10 | 160 | >16 |
| BQ (adult/ F) | 01-2012 | Yamagata | -85 | 265 | <10 | <10 | NA | <10 | <10 | NA |
| BR (children/ F) | 02-2012 | Yamagata | -78 | 300 | <10 | <10 | NA | <10 | 20 | >2 |
| BS (children/ M) | 12-2011 | Yamagata | -61 | 306 | <10 | 40 | >4 | <10 | 40 | >4 |
| BT (children/ M) | 12-2011 | Yamagata | -61 | 306 | <10 | <10 | NA | <10 | 40 | >4 |
| BU (adult/ F) | 02-2012 | Yamagata | -103 | 264 | <10 | <10 | NA | <10 | 20 | >2 |
| BV (children/ F) | 03-2012 | Yamagata | -132 | 225 | 40 | 80 | 2 | <10 | 80 | >8 |
| BW (children/ M) | 01-2012 | Yamagata | -70 | 280 | <10 | <10 | NA | <10 | <10 | NA |
| BX (adult/ F) | 01-2012 | Yamagata | -70 | 280 | <10 | 80 | >8 | <10 | <10 | NA |
| BY (adult/ F) | 04-2012 | Yamagata | -187 | 191 | <10 | <10 | NA | <10 | 20 | >2 |
| BZ (children/ M) | 03-2012 | Yamagata | -133 | 217 | <10 | <10 | NA | <10 | <10 | NA |
| CA (children/ M) | 09-2013 | Yamagata | -281 | 55 | <10 | 10 | >1 | <10 | <10 | NA |
| CB (children/ M) | 09-2013 | Yamagata | -328 | 50 | <10 | 40 | >4 | <10 | 40 | >4 |
| CC (children/ M) | 01-2014 | Yamagata | -72 | 96 | 40 | 40 | 1 | <10 | 10 | >1 |
| CD (adult/ M) | 04-2014 | Yamagata | -146 | 29 | <10 | 10 | >1 | <10 | <10 | NA |
| CE (adult/ F) | 04-2014 | Yamagata | -146 | 29 | <10 | <10 | NA | <10 | <10 | NA |
| CF (children/ M) | 02-2014 | Yamagata | -111 | 275 | <10 | <10 | NA | <10 | <10 | NA |
| CG (children/ F) | 04-2014 | Yamagata | -156 | 26 | 10 | 160 | 16 | <10 | 160 | >16 |
| CH (children/ M) | 03-2014 | Yamagata | -109 | 241 | <10 | <10 | NA | <10 | <10 | NA |
| CI (adult/ M) | 03-2014 | Yamagata | -109 | 241 | 20 | <10 | < -2 | <10 | 40 | >4 |
| CJ (children/ M) | 04-2014 | Yamagata | -167 | 211 | <10 | <10 | NA | <10 | 40 | >4 |
