# Supplementary material for: Variation by lineage in serum antibody responses to influenza B virus infections
Source: PLoS One. 2020 Nov 9;15(11):e0241693. doi: 10.1371/journal.pone.0241693 (PMC7652285; doi:10.1371/journal.pone.0241693)
Supplement: S2 Table — The time for antibody titer reaching the peak and the period for short-term waning rate were fixed to be 4 weeks and 6 months after infection in the model. This table compared the estimates when the time for antibody titer reaching the peak was 2 weeks and 8 weeks, and when the period for short-term waning rate was 3 months and 9 months. (DOCX) [file pone.0241693.s002.docx]

**Table S2. Sensitivity analysis on different time for antibody titer reaching the peak and different period for short-term waning rate.** The time for antibody titer reaching the peak and the period for short-term waning rate were fixed to be 4 weeks and 6 months after infection in the model. This table compared the estimates when the time for antibody titer reaching the peak was 2 weeks and 8 weeks, and when the period for short-term waning rate was 3 months and 9 months.

| Estimates (95% CrI) |  |  |  |  |  |  |  |  |
| --- | --- | --- | --- | --- | --- | --- | --- | --- |
| Time for antibody titer reaching the peak (weeks) | | | | 4 | 2 | 8 | 4 | 4 |
| Period for short-term waning rate (months) | | | | 6 | 6 | 6 | 3 | 9 |
| Lineage of infection | Mean fold-rise | Age group | Pre-infection titer |  |  |  |  |  |
| B/Victoria | B/Victoria | Children | <10 | 19.5 (5.6, 48.0) | 20.7 (5.8, 53.5) | 17.2 (5.3, 39.0) | 24.0 (5.9, 72.4) | 18.0 (5.4, 41.3) |
|  |  | Children | 40 | 3.6 (1.0, 15.8) | 3.7 (1.0, 16.2) | 3.3 (1.0, 14.2) | 3.9 (1.0, 17.9) | 3.5 (1.0, 15) |
|  |  | Adults | <10 | 3.0 (1.0, 14.2) | 3.2 (1.0, 16.1) | 2.7 (1.0, 12.4) | 3.9 (1.0, 19.9) | 2.8 (1.0, 12.9) |
|  |  | Adults | 40 | 1.4 (1.0, 3.8) | 1.4 (1.0, 4.0) | 1.3 (1.0, 3.4) | 1.5 (1.0, 4.6) | 1.3 (1.0, 3.7) |
|  | B/Yamagata | Children | <10 | 2.8 (1.0, 7.7) | 2.9 (1.0, 8.3) | 2.6 (1.0, 6.8) | 3.1 (1.1, 9.5) | 2.7 (1.0, 7.2) |
| B/Yamagata | B/Yamagata | Children | <10 | 20.0 (6.4, 50.4) | 21.5 (6.5, 57.3) | 17.5 (6.1, 40.4) | 24.8 (6.8, 75.2) | 18.6 (6.3, 45.6) |
|  |  | Children | 40 | 8.7 (1.9, 24.7) | 9.4 (1.9, 27.7) | 7.3 (1.8, 19.8) | 10.8 (2.0, 34.9) | 8.1 (1.9, 22.9) |
|  |  | Adults | <10 | 4.5 (1.0, 21.8) | 5.0 (1.0, 25.5) | 3.5 (1.0, 16.7) | 6.4 (1.0, 33.3) | 4.0 (1.0, 19.1) |
|  |  | Adults | 40 | 2.9 (1.0, 12.2) | 3.2 (1.0, 13.5) | 2.4 (1.0, 9.3) | 3.9 (1.0, 17.6) | 2.7 (1.0, 11.0) |
|  | B/Victoria | Children | <10 | 3.7 (1.3, 9.0) | 3.8 (1.3, 9.7) | 3.3 (1.3, 7.4) | 4.3 (1.3, 12.7) | 3.4 (1.3, 7.9) |
