# Supplementary material for: Variation by lineage in serum antibody responses to influenza B virus infections
Source: PLoS One. 2020 Nov 9;15(11):e0241693. doi: 10.1371/journal.pone.0241693 (PMC7652285; doi:10.1371/journal.pone.0241693)
Supplement: S3 Table — This table showed the proportion of observation which lied inside the 95% credible intervals of the post-infection titer by lineage of PCR-confirmed infection and lineage titer. (DOCX) [file pone.0241693.s003.docx]

**Table S3. Model adequacy for fitting the data.** This table showed the proportion of observation which lied inside the 95% credible intervals of the post-infection titer by lineage of PCR-confirmed infection and lineage titer.

| % | Post-titer in main analysis | | Post-titer in sub-analysis | |
| --- | --- | --- | --- | --- |
| PCR result | B/Victoria | B/Yamagata | B/Victoria | B/Yamagata |
| Overall | 96 | 98 | 95 | 97 |
| B/Victoria | 100 | 100 | 100 | 100 |
| B/Yamagata | 96 | 100 | 100 | 100 |
| Negative | 94 | 95 | 92 | 95 |
